# Innovative Detection of Testosterone Esters in Camel Hair: Unravelling the Mysteries of Dromedary Endocrinology

**DOI:** 10.3390/molecules29010097

**Published:** 2023-12-22

**Authors:** Iltaf Shah, Muhammad K. Hakeem, Aysha Alraeesi, James Barker

**Affiliations:** 1 Department of Chemistry, College of Science, UAE University, Al Ain P.O. Box 15551, United Arab Emirates; 700039966@uaeu.ac.ae (M.K.H.); 201209669@uaeu.ac.ae (A.A.); 2 School of Pharmacy and Chemistry, Kingston University, Kingston upon Thames KT1 2EE, UK; j.barker@kingston.ac.uk

**Keywords:** testosterone, testosterone esters, racing camels, hair analysis, LC–MS/MS, liquid chromatography mass spectrometry

## Abstract

**Introduction:** Doping and steroid use represent a serious threat to animal health and can even lead to their untimely and painful death. However, doping is an acute problem in today’s animal racing world, particularly in camel racing. Testosterone and its ten esters (benzoate, valerate, isocaproate, hexahydrobenzoate, decanoate, undecanoate, laurate, enanthate, cypionate, and caproate) are of utmost importance, because when they are administered to animals it is difficult to measure them efficiently. The levels of testosterone and its esters in camels and other animals are typically determined using urine and blood tests. The aim of this study was to develop and validate a liquid chromatographic–mass spectrometric (LC-MS/MS) method to determine testosterone esters in camel hair, and to apply the validated method to determine testosterone esters in collected samples. To our knowledge, this is the first report of such research. **Results and Discussion:** The levels of testosterone and its ten derivatives, along with the cortisol-D4 internal standard, were optimised for LC–MS/MS analysis; however, only testosterone along with its seven esters (namely benzoate, valerate, isocaproate, hexahydrobenzoate, decanoate, undecanoate and laurate) could be validated in camel hair. Only five testosterone esters could be determined in camel hair samples; the concentrations were obtained as 10.5–14.9 pg/mg for valerate (in three camels), 12.5–151.6 pg/mg for hexahydrobenzoate (in six camels), 4.8–32.1 pg/mg for laurate (in five camels), 5.1 pg/mg decanoate (in one camel), and 8.35–169 pg/mg for testosterone (in all 24 camels). Interestingly, the three racing camels displayed high concentrations of testosterone (59.2–169 pg/mg, all three camels), laurate (4.8–14.5 pg/mg, two camels), hexahydrobenzoate (116 pg/mg, one camel), decanoate (5.1 pg/mg, one camel), and valerate (11.7 pg/mg, one camel). **Methods:** Camel hair samples were collected from 21 non-racing dromedary camels along with three racing camels in Al Ain, UAE; these were decontaminated, pulverised, sonicated, and extracted prior to analysis. An LC–MS/MS method was employed to determine the levels of testosterone esters in the hair samples. **Conclusions:** This novel camel-hair test procedure is accurate, sensitive, rapid, and robust. The findings reported in this study could be significant to evaluate racing camels for suspected doping offenses. Further controlled testosterone supplementation studies are required to evaluate individual esters’ effects on camel health and diseases and on performance enhancement levels. This new hair test could promote further studies in doping control, toxicology, and pharmacology, as well as having other clinical applications relating to camel health, injury, and disease.

## 1. Introduction

### 1.1. Overview

Steroid misuse is an acute global problem involving both humans and animals [1]. Different types of drug therapy have been adopted to treat animals and enhance their performance, or to treat an injured animal such that it is more likely to win a competition [2]. In particular, steroids and anabolic-androgenic steroids (AAS) are used by animal—especially camel—owners to enhance the competitiveness of the camels. Steroid usage has also expanded from elite athletic circles into high-school athletic programs. Steroid use has been accused of providing an unfair advantage and damaging the true talent of a competitor. Performance-enhancement drugs are considered to be attractive by younger generations, and this may increase the usage of these drugs in the future [3]. Performance-enhancing drugs are considered unethical and a form of cheating; hence, they are illegal and have been prohibited by the World Anti-Doping Agency (WADA). In most countries, doping has been strictly prohibited as part of curbing the illegal use in the sporting world and promoting healthy competition. Doping has been prevalent for many years in sports, with no positive indication of being diminished [4]. Recent years have seen a significant increase in awareness and research into various aspects of doping. Awareness campaigns are necessary for educating current generations about the harmful effects of doping on society and the health of humans and animals.

### 1.2. Animal Racing

Animal racing has developed into more than just a sport; it is now a fascinating fusion of competitive zeal, cultural tradition, and, as we will see, a special scientific window into the field of endocrinology. The spectacle of racing animals, whether camels, horses, or other majestic creatures, captivates spectators and shapes communities in various cultures and countries. We will explore the field of animal racing by focussing on camel racing to help us understand the enigmas surrounding dromedary endocrinology. Camel racing is widely popular in Gulf countries; in Dubai, it is a huge attraction for visitors from across the world and for Emiratis. Special efforts have been made to maintain the core traditions of the game.

The extravagant nature of the competition and the lucrative prizes available often compel the trainers to cheat. The camels are subjected to doping procedures to increase their performance and to gain a competitive advantage. The sport has been considerably updated in recent years, to ensure fair competition [5]. Doping is generally illegal across the globe [6]. In the UAE specifically, rigid policies have been introduced to restrain doping practices in the region. Three types of methods are used to dope camels: liniments, oral drugs, and injections. Certain drugs (e.g., ammonium chloride and furosemide) are used to treat medical conditions in animals; these are legal, and therapeutic use exemptions can be obtained for them [7].

A number of trainers and veterinarians have been charged for administering performance-enhancing drugs (PEDs) to animals. Recently, the organisers of the Dubai Camel Racing Festival announced that they have made significant improvements in the anti-doping testing procedures. Doping can be detected by testing the hair, urine, and saliva samples of a person or animal. Anabolic steroids present in human hair have been detected using novel, highly specific, sensitive, and reliable liquid chromatography–tandem mass spectrometry (LC-MS/MS) methods [8]. Hair analyses are used as a complementary test to blood and urine analyses. Recently, hair analysis has been implemented to identify chronic drug use or long-term drug doping [9]. Thus, hair represents the best method for identifying competition doping.

### 1.3. Hair Analysis

Hair analysis is a safer, non-invasive test procedure than other methods [10], and hence no harm is given to the animals. Hair samples are easy to store and transport, and the sample quality does not rapidly degrade [11]. Hair analysis presents a negligible risk of infection during collection, reducing the risks of cross-contamination and tampering. The average hair growth is 1–1.5 cm per month (in humans and animals), and the larger strands contain more retrospective information regarding drug intake than the short-term usage information found in urine or blood samples. Occasionally, results that detect and confirm steroid usage can be false positives, owing to the consumption of foods containing undeclared substances. Animal owners use steroids to increase the animal strength, body mass, and aggressiveness; they can also help to shorten the recovery time between different races. In 1974, the International Olympic Committee added doping agents to the list of prohibited substances, owing to their effects on athlete performances [4]. In sports—where steroid usage is popular among animal owners—urine specimen analyses fail to clearly distinguish between single exposures or chronic use. Hair analysis provides a larger detection window and can identify drug users who try to cheat the test by diluting their urine sample or abstaining from drugs for a few days before the test. Hence, hair testing or analysis can be seen as a more modern method that offers significant advantages such as increased accuracy and tamper resistance. Thus, hair analysis is beneficial and presents a negligible risk of infection, cross-contamination, or tampering [12].

### 1.4. Testosterone, an Anabolic Steroid

Anabolic steroids (more correctly referred to as anabolic-androgenic steroids) are steroidal androgens that contain normal androgens (e.g., testosterone) along with synthetic androgens, which are physically connected and have the same effects as testosterone. Diverse side effects can arise when anabolic steroids are distorted. The side effects of anabolic steroids can include severe acne, hair loss, oily skin and hair, liver diseases (e.g., cysts and liver tumours), heart diseases (e.g., strokes or heart attacks), Q1A diseases, altered moods, increased violence, suicidal tendencies, depression, and changes to fat and other blood phosphatides. Some people who inject anabolic steroids may lack sterile injection methods, or they may share unclean needles with other people; this places them at risk of life-endangering viral contagions (e.g., HIV and Hepatitis B and C). Furthermore, animal models indicate that anabolic steroids destroy the immune system, which can exacerbate infections [13]. Furthermore, steroidal dietary supplements can be converted into testosterone or other androgenic mixtures in the body. The decrease in testosterone leads to reduced sperm formation and testicle sizes. Anabolic steroids can also perform hormone coordination, increasing the possibility of testicular cancer [14].

**Figure 1:**
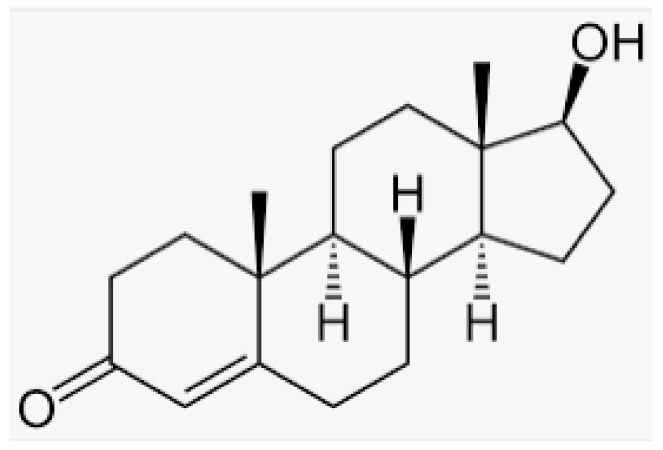
Chemical structure of testosterone [4].

Testosterone (chemical structure shown in Figure 1) is involved in the growth of muscle volume and strength. It stimulates the growth of neurotransmitters that promote tissue and growth hormone development. Increasing testosterone levels promote the development of the testicles, pubic hair, and penis [15].

Testosterone and other anabolic-androgenic steroids improve athletic performance. Many of the testosterone products approved for therapy nowadays are testosterone esters. Esters have multiple functions and are mixed with testosterone to extend their lifespan and bulk manipulability. When infused, pure testosterone has a short half-life, enduring in the body for just a few hours [16]. Short-term testosterone usage may also have cryogenic impacts, as exhibited by improved maximal bench-press power [15]. The testosterone hormone is affected by genomic and non-genomic pathways; the cytoplasmic androgen receptors bind with the biologically inactive testosterone in an unbound state. Dihydrotestosterone also similarly activates the androgenic receptor (AR). The binding with testosterone stimulates the mobilisation of Ca^2+^, which increases its level in the intercellular spaces [17,18,19]. The testosterone receptors associated with the membrane are linked with the G-proteins, which are in turn associated with the phospholipase. In certain cases, aggressive behaviour is expressed as a tendency to break norms. Higher levels of endogenous testosterone are directly associated with a higher tendency to take risks and face challenges. Androgen activity is dependent on the level of testosterone, which is in turn affected by various other factors [20,21] and this is true in other species as well. Moreover, testosterone affects the morphology of the body and other characteristics. Mental rotation is a key phenomenon investigated in the context of gender-specific effects of testosterone.

### 1.5. Studies Conducted to Identify Steroids in Animal Hair

Table 1 presents several studies conducted to identify steroids in animal hair using various advanced analysis techniques.

The above table gives a comparison of the methods published for analysis of hair in different categories of cattle and other animals. The table compares the following important details. Detection methods used, type of mobile phase employed, types of steroids detected, column used for chromatographic separation, and extraction of steroids from hair discussed, along with their respective LOD and LLOQ where possible. Overall, the LCMS/MS and GCMS methods have been more sensitive, reliable, and reproduceable. EIA and RIA methods in general suffer from cross-reactivity issues along with poor sensitivity in most cases. Methanol and formic acid have been widely used as solvents as thr mobile phase in the LCMS/MS method. Often a reverse phase C18 column has been used for chromatographic separation. Mostly, a combination of liquid–liquid extraction is followed by solid phase extraction, which makes the extraction process very labour-intensive. The extraction method we report here is much simpler, less laborious, and it is also very robust and reproduceable.

## 2. Results and Discussion

The chromatographic conditions facilitated the separation, identification, and quantitation of testosterone esters; this was achieved using a reversed-phase column. Using chromatographic separation, this method could effectively separate the major testosterone esters and their metabolites. Although these major testosterone esters and metabolites have similar structures and masses, a difference was observed between them in terms of their retention times, as shown in Table 2 below. The combination of HPLC and MS/MS components provides a powerful tool for detecting and confirming the presence of drugs in complex biological matrices. This technique offers high specificity and sensitivity, and it is widely applied for the simultaneous identification of testosterone and other steroids.

**Figure 2:**
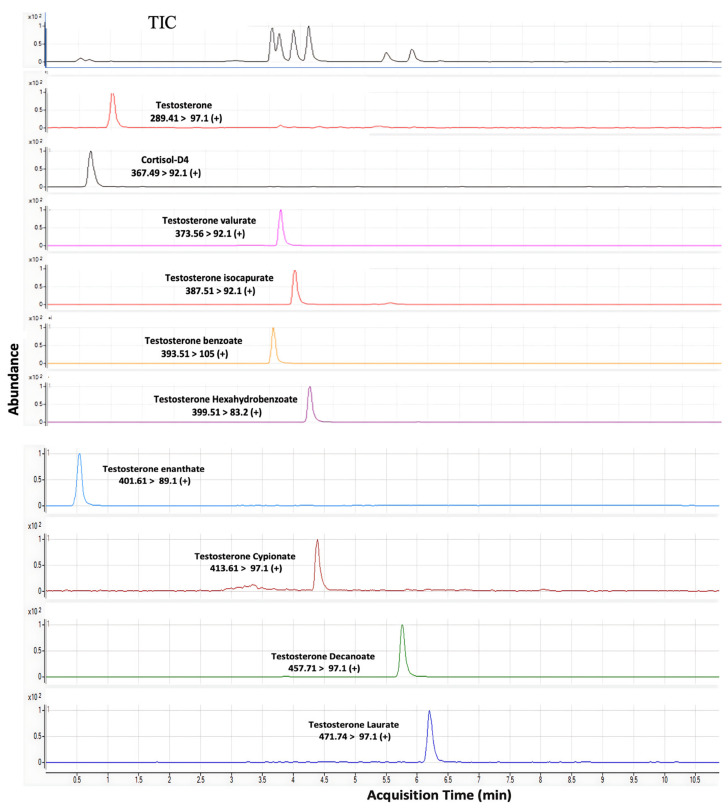
Representative chromatogram of testosterone esters separated by LC–MS/MS.

The most popular versions of the LC–MS method are the tandem mass spectrometry and “multiple reaction monitoring” (MRM) modes. These approaches use tandem mass spectrometers to detect a specific product ion generated from a precursor ion (the parent compound) under a given set of fragmentation conditions. Figure 2 shows the chromatograms of esters of testosterone at their specific retention time. The monitoring of the precursor to product ion facilitates the specific and accurate determination of given analytes, even if they are not chromatographically resolved in the liquid chromatography stage of the LC-MS/MS method. Initial experiments were conducted using pure analytical standards to identify the precursor and product ions for specific analytes. Their structures and abbreviations, the mass-to-charge ratios (*m*/*z*) for the precursor and products ions, and the fragmentor voltage and collision energies values determined for the various analytes are summarised in Table 3. According to Table 3, although testosterone esters exhibit almost identical retention times and polarities, their masses differ; thus, they are separatable via their mass-to-charge (*m*/*z*) ratios. The polarities of these esters are slightly different; hence, they can be separated by chromatography, using the aforementioned reversed-phase column. The chromatographic conditions established in this study facilitated the separation, identification, and quantitation of analytes with different retention times.

Table 3 shows the analyte names, the mass of all testosterone esters, the precursor and product ions, and the collision energies of all testosterone esters. The precursor and product ions together are referred to as MRM transitions.

### Camel Sample Results

**Figure 3:**
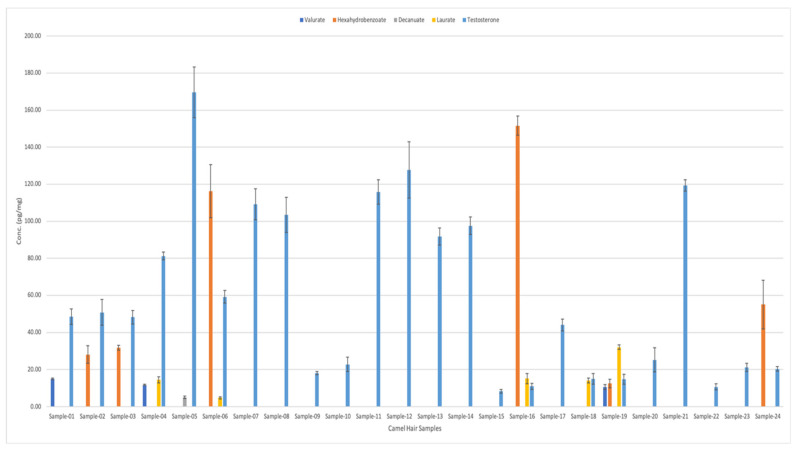
Analysis of the chosen testosterone esters in camel hair samples, obtained using LC–MS/MS.

Figure 3 shows the types and quantities of testosterone esters found in 24 camel hair samples. Samples number 3, 4, and 5 were from racing camels. Interestingly, testosterone was present in all 24 samples. It exhibited the highest concentrations for all samples (ranging from ~10 pg/mg to 185 pg/mg). This was followed by the hexahydrobenzoate, which was present in six different hair samples in various concentrations (from 12.6 pg/mg to 151 pg/mg).

**Figure 4:**
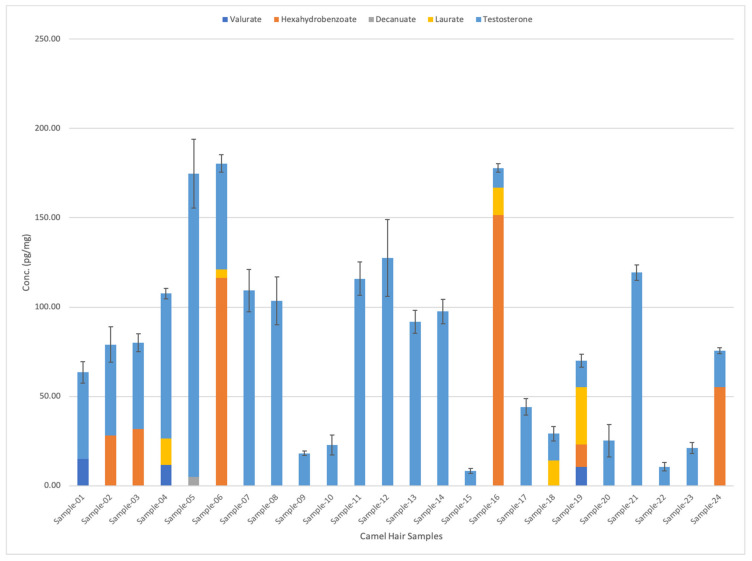
Comparison between testosterone esters concentrations in 24 camel hair samples. The standard errors of the mean are represented by the error bars in the graph.

On the other hand, valerate appeared in three different samples with average concentrations of 10.5, 11.7, and 14.98 pg/mg, respectively (as shown in Figure 3). Meanwhile, laurate analytes were detected in five different samples in varying concentrations: 4.83, 14.1, 14.4, 15.1, and 32.1 pg/mg. Finally, decanoate was only detected in Sample 5 (from racing camel), with a concentration of 5 pg/mg. Table 4 shows the detailed comparison of different testosterone esters in camel hair samples. Figure 4 shows the graphical representation of testosterone esters concentrations in 24 camel hair samples.

Testosterone, its ten esters, and cortisol-D4 internal standard were optimised for LC–MS/MS analysis. This was performed by running the working solutions by selecting the best precursor and product ions in multiple reaction monitoring (MRM) mode using the Agilent LC-MS/MS optimiser MassHunter software (version B.07.01) application. Nothing was administered to the camels. However, only eight esters—namely, testosterone, benzoate, valerate, isocaproate, hexahydrobenzoate, decanoate, undecanoate, and laurate—could be verified in camel hair. Only five esters of testosterone could be determined in camel hair samples, with concentrations of 10.5–14.9 pg/mg for valerate (in three camels), 12.5–151.6 pg/mg for hexahydrobenzoate (in six camels), 4.8–32.1 pg/mg for laurate (in five camels), decanoate 5.1 pg/mg (in one camel), and 8.35–169 pg/mg for testosterone (all 24 camels). Interestingly, the three racing camels (Samples 3, 4, 5) displayed high concentrations of testosterone (59.2–169 pg/mg, all three camels), laurate (4.8–14.5 pg/mg, two camels), hexahydrobenzoate (116 pg/mg, one camel), decanoate (5.1 pg/mg, one camel), and valerate (11.7 pg/mg, one camel). The reason for this is to increase their performance beyond their natural abilities as well as for the treatment of injuries.

## 3. Materials and Methods

### 3.1. Materials

Testosterone, testosterone isocaproate, testosterone enanthate, testosterone benzoate, testosterone cypionate, testosterone undecanoate, testosterone valerate, testosterone laurate, testosterone caproate, testosterone hexahydrobenzoate, and testosterone undecenoate were all purchased from Sigma Aldrich (Taufkirchen, Germany); the cortisol-D4 internal standard was supplied by Labco Ltd., UAE. Methanol, hexane, formic acid, deionised water, and LC–MS-grade water and acetonitrile were purchased from Emirates Scientific and Technical Supplies L.L.C. (ESTS) (Dubai, United Arab Emirates). An Ascentis Express F5 column (150 mm × 2.1 mm × 2.7 μm), plastic bags, glass test tubes (5 mL), and glass Pasteur pipettes were received from Gomet (Al Ain, United Arab Emirates). Millex Samplicity TM Filters, 0.20 μm hydrophilic polytetrafluoroethylene (Merck Millipore, Carrigtwohill, Ireland, cat. no. SAMPLG001), and 2 mL glass chromatographic vials with caps (2 mL) were purchased from Emirates Scientific Supplies Ltd., Abu Dhabi, United Arab Emirates.

#### 3.1.1. Preparation of Standard Solutions

The stock solutions of each testosterone metabolite and internal standard were prepared in methanol with a concentration of 1 mg/mL. All stock solutions were kept and stored in amber vials at −20 °C. The working standards solution for the testosterone esters and internal standard were prepared via serial dilution of stock solutions in methanol to obtain the desired concentrations.

#### 3.1.2. Collection of Hair Samples

The hair samples of 24 camels were collected from a farm in Al Ain, UAE with length 1.5–5 cm and width of about a thumb thickness. The hair samples were cut specifically from the hump (i.e., the large lump on the camel where fat is stored). This was performed with particular attention, to avoid any damage to the skin. All hair samples were kept individually in clear, labelled plastic bags at room temperature. The study protocols were approved by the UAEU research ethics committee. Blank hair was selected from one of these 24 camel hair samples. The selection of blank hair war carried out by collecting six different lots of presumed healthy drug-free camels. Samples from male and female camels, with different hair colours (light brown to dark brown), were also included in this evaluation. Selectivity and interference from the matrix were determined using the extraction of six lots of blank camel hair samples and running them on the LC-MS/MS method. No interfering peaks were detected at the retention time of any of the testosterone esters in any of the samples evaluated. One out of the six samples that was the most clean blank was selected for preparation of calibration curve and quality controls.

#### 3.1.3. Hair Sample Extraction Method

The hair samples were washed with methanol/water and then dried down under a gentle stream of N_2_, then about 100 mg hair was ground to a powder using a Mini-ball mill (Pulverisette 23, Fritsch, Germany) operating at 50 oscillations per second for 15 min. Then, 20 mg of ground hair was carefully weighed out into 25 mL labelled borosilicate culture tubes; 100 μL of methanol was added to each hair sample. Then, 25 μL of internal standard cortisol-d4 (concentration: 1 ng/mL) was added to all hair samples after grinding except the blank matrix one, and the samples were incubated in an ultrasonic bath (Branson 5800, Danbury, CT, USA) at 40 °C for 120 min. Samples were incubated in a sonic bath at 50 Hz, 55 °C for 240 min before being removed. Next, the samples were vortexed for 1 min at room temperature, and 1000 μL hexane was added to the mixture for extraction. Samples were vortex-mixed again for 1 min at room temperature.

Next, the tubes were centrifuged at 1300× *g* for 20 min (Beckman TJ-6, Beckman, UK). After centrifugation, the top organic layer was separated into new glass test tubes using Pasteur pipettes. Then, the extract was aliquoted into tubes and dried under a stream of nitrogen gas at 40 °C (Techne, Bibby Scientific, Burlington, VT, USA). The ground samples were then extracted using 5 μL of high-performance liquid chromatography (HPLC)-grade methanol. Finally, the dry extract was reconstituted with 100 μL of methanol and placed in 2 mL HPLC vials with glass inserts, to be injected into the LC-MS/MS system.

#### 3.1.4. Liquid Chromatography and Mass Spectrometry

The chromatographic separation of testosterone esters was performed using an Agilent 1260 HPLC system on a reversed-phase column (Zorbax Eclipse Plus C18 (Agilent Technology, Santa Clara, CA, USA)) with a particle size of 2.1 mm, an inner diameter of 1.8 μm, and a length of 50 mm. The chromatographic column was maintained at a constant temperature of 35 °C and a constant mobile phase flow rate of 0.4 mL/min. The injection volume for analysis into the LC–MS/MS system was 4 μL. The sample contamination was minimised by a wash program that rinsed the needle with a methanol/water (75/25, *v*/*v*) mixture. Two mobile phases were used during analysis: Mobile Phase A consisted of 35% acetonitrile and 35% methanol, 30% water, and 2 mL of dissolved formic acid (all LC–MS grade); meanwhile, Mobile Phase B consisted of 50% water, 50% acetonitrile, and 2 mL of dissolved formic acid (all LC–MS grade). Optimal testosterone metabolite chromatography conditions were achieved using a binary mobile phase gradient pump, where gradient elution was conducted as follows: Mobile Phase A was held at 100% whilst B was kept at 0.0%; then, this was changed to 100% for B and 0% for A over 30 s. The gradient was held at this ratio for 7.5 min. Between 7.5 and 8 min it was brought back to 100% A and 0% B, and the same gradient was then maintained for 4 min. The mass spectrometry analysis was performed using an Agilent 6420 Triple Quadrupole Mass Spectrometry system in the positive electrospray ionisation (ESI+) mode. The electrospray voltage was set at 4000 V, the ion source gas 1 (a desolvation gas consisting of 99.9% nitrogen) pressure was set at 20 psi, the ion source gas 2 (a nebuliser gas consisting of nitrogen) was set at 45 psi, and the drying gas (N_2_) flow was 8 L/min at 325 °C. The HPLC system consisted of a pump, auto-sampler, column oven, and degasser controlled by Aglient MassHunter™ software (version B.07.01). Moreover, the auto-sampler component had a high injection speed, and the system could use multiple solvents simultaneously. The Tandem Mass Spectrometer system with a newly developed UF-Qarray ion guide increased the LC-MS/MS sensitivity by enhancing the ion signal intensity and reducing noise, providing a new level of speed and responsiveness.

#### 3.1.5. Data Analysis

The standard addition method was used for analysis of endogenous testosterone, while all other esters were analysed by spiking blank camel hair (known to be free from any exogenous testosterone esters). The selection of blank hair was carried out by collecting six different lots of presumed healthy drug-free camels. Samples from male and female camels were also included in this evaluation. Selectivity and interference from the matrix were determined using the extraction of six lots of blank camel hair samples and running them on the LC-MS/MS method. No interfering peaks were detected at the retention time of any of the testosterone esters in any of the samples evaluated. One out of the six samples that was the most clean blank was selected for preparation of calibration curve and quality controls.

The extraction efficiency was evaluated by determining the recovery percentage. The recovery was implemented by preparing two sets of quality controls, where each set consisted of three levels of quality control (QCH, QCM, QCL) and each level was prepared six times. In the first set, the quality control samples for QCH, QCM, and QCL were spiked with methanol; then, the internal standard was added to all QC samples; however, the extraction method was not performed; hence, this set contained unextracted QC samples. In the second set, the quality control samples for QCH, QCM, and QCL were spiked with blanks; then, the internal standard was added to all QC samples; next, extraction was performed; and as a result, this set included extracted QC samples. The area under the normal peak in the chromatogram for the extracted QC samples was compared with that for unextracted QC samples; then, the recovery percentage was calculated. In all six sets, the quality control samples for QCH, QCM, and QCL were spiked with blank hair. In the first set, the internal standard was added to all QC samples; then, extraction was performed. In the second set, the internal standard was added to all QC samples; then, the extraction was performed using 1 mL hexane. The specificity experiment was executed by analysing six blank camel hair samples; then, each sample chromatogram was compared with other samples, to detect interferences (e.g., matrix components and impurities). Samples were vortex-mixed again for 1 min. The specificity experiment was performed by analysing four blank hair samples; then, the chromatogram of each sample was compared with that of other samples to detect interferences (e.g., matrix components and impurities).

#### 3.1.6. Method Validation

Validation was performed in accordance with the US Food and Drug Administration (FDA) guidance, which suggested that the bioanalytical method should be developed and validated by optimising the conditions and procedures for the extraction and detection of the analyte and its esters [29]. The method validation parameters (i.e., intra- and inter-day precision, intra- and inter-day accuracy, recovery, linearity, and specificity) were calculated by analysing six QC samples at three different concentration levels: quality control high (QCH), quality control medium (QCM), and quality control low (QCL). The QC values were used to calculate the percentages of intra- and inter-day precision, using the following equation: % CV = (*Standard deviation*) ÷ (*mean*) × 100.

The percentages of Intra- and inter-day accuracy were calculated using the following equation:

% Accuracy = (*mean value*) ÷ (*nominal value*) × 100.

The extracted and unextracted QC values were used to calculate the percentage of absolute recovery, using the following equation: % Recovery = (*mean extracted QC values*) ÷ (*mean unextracted QC values*) × 100.

The instrument’s sensitivity toward testosterone esters was measured using the lower limit of quantitation (LLOQ); this was determined by comparing the peak intensities for the lowest analyte concentration against the noise level of the chromatogram. The lowest analyte concentration should exhibit a response three times larger than the background or noise level. The linear range of several testosterone esters was 0.9–1000 ng/mL, with a very good regression value (linearity). A low LOD was determined from the lowest concentration of testosterone esters detectable by the instrument. The LOD for this method was 0.48.

The method validation parameters were identified within an acceptable range for each QC level. For example, the percentages of the variation and accuracy coefficients indicate that the method used to analyse testosterone esters offers good intra- and inter-day precision and accuracy. Moreover, the method demonstrates a strong recovery percentage, which means that the extraction method is efficient at extracting testosterone esters from hair samples, as shown in Table 5. Moreover, the table shows the intra- and inter-day precision and accuracy results of six QC samples for each QC level. Furthermore, the values of percentage recovery were calculated by comparing the areas under the normal curve in the chromatogram for the extracted and unextracted QC samples.

## 4. Conclusions

Doping is a serious threat to the health of animals, and it can have dire health consequences upon the person or animal exposed to it. In this study, an effective method was developed for the determination of testosterone metabolite in camel hair; it is highly sensitive, robust, and reproducible. For extraction, hexane—a low-toxicity solvent—was used. The new method shows high sensitivity and high reproducibility. By using the reversed-phase column Zorbax Eclipse Plus C18, all testosterone esters could be separated. From the linearity results, the quantification method was found suitable for the analysis of camel hair samples. The authors believe that this innovative camel hair test could be used to complement blood, saliva, and urine tests for the detection of testosterone esters in camels. The new hair test could be used to evaluate long-time testosterone doping for injury recovery or performance enhancement. The testosterone hair test for camels could be further extended to cover segmental analysis of camel hair, to detect long-term exposure. The bioanalytical method was fully validated according to the US Food and Drug Administration (US-FDA) guidelines for the optimisation of procedures and conditions to extract and detect esters. Moreover, the parameters of the bioanalytical method (e.g., its accuracy, linearity, specificity, precision, and recovery) ensure the suitability of this method. This novel camel hair test is accurate, sensitive, rapid, and robust. The findings reported in this study could be significant for evaluating racing camels for suspected doping offenses, as well as for injury and disease control and long-term exposure. Further controlled testosterone supplementation studies are necessary to evaluate individual testosterone metabolite effects on camel health, diseases, and performance enhancement. This new hair test could facilitate further studies in doping control, toxicological, pharmacology, and other clinical fields.

## Figures and Tables

**Table 1:** Methods used for determining different steroids in the hair of domestic and wild species.

Year	Animal	Detection Method and Mobile Phase	Steroid Detected and Column Used	Extraction Method	LOD	LLOQ	Reference
2017	Domestic cattle breeds	QuadrupoleLC–MS/MS. Mobile phase solvent A consisted of ammonium formate (200 mM) with 0.1% formic acid and eluent B consisted of ammonium formate (200 mM) in methanol.	Cortisol Column: 50 × 2.10 mm 2.6 μm C18 column	20 mg hair snippets were extracted in 3 mL methanol for 16 h overnight in an ultrasonic bath at 55 °C	Regrown hair/Hair from unshorn area 0.2 pg/mg/ 0.1 pg/mg	1 pg/mg/ 0.5 pg/mg	[22]
2015	House rat/mouse	LC–MS/ MS The mobile phase was a mixture of methanol and water (90:10 *v*/*v*) containing 2.0 mM ammonium acetate, which was filtered through a microporous membrane (0.22 μm) before use	Cortisol Column: 5 μm, 150 × 4.6 mm, Platisiltm ODS-C18	20 mg of hair pieces was incubated in 1 mL of methanol for 24 h followed by the solid phase extraction using (SPE) C18 column	House rat/House mouse 0.5/1.25 pg/mg	1.25/2.5 pg/mg	[23]
2015	Domestic cattle: Holstein	Enzyme Immunoassay (EIA)	Cortisol	20 mg was taken 1 mL methanol added and incubated at 100 rpm at 50 °C	1 pg/mg	2 pg/mg	[24]
2009	Bovine hair	LC–MS/MS Mobile Phase: solvent A, water–acetonitrile–methanol–formic acid (300:350:350:20, *v*/*v*/*v*/*v*); solvent B, acetonitrile–methanol–formic acid (500:500:20, *v*/*v*/*v*))	Estradiol benzoate (EB), testosterone cypionate (TC) and testosterone decanoate (TD)Column: Waters acquity UPLC BEH C18 analytical column of 100 × 2.1 mm and 1.7 μm particle size	200 mg hair was digested with the 2 mL reduction agent tris(2-carboxyethyl) phosphine hydrochloride (TCEP) for 1 h, followed by extraction using SPE C18 cartridges	2 pg/mg for all	5 pg/mg for all	[25]
2009	Himalayan Tahr	Radioimmunoassay (RIA)	Testosterone	50 mg hair was collected and incubated with 3 mL of 1 M NaOH for 40 min, at 38 °C. A 300 μL aliquote was extracted with 5 mL of diethyl ether	0.9 pg/mg	Not reported by authors	[26]
2007	Domestic horse (Equus ferus caballus)	GC–MS/MS The following temperature ramp was used: 150 °C (1 min) to 320 °C at 18 °C/min. The temperature of the source was 280 °C and the transfer line was set to 300 °C.	Testosterone Column: silica capillary column DB5MS, 30 m × 0.25 mm i.d. × 0.25 μm	Tail hair samples (100 mg) were dissolved in 1 mL of sodium hydroxide for 15 min at 95 °C. Followed by diethyl ether extraction and a SPE Isolute C18 cartridges eluted with methanol	1 pg/mg	2 pg/mg	[27]
2018	Dogs	LC-MS/MS Mobile phase: Solvents were 0.1% formic acid: 0.1% ammonium formate (1:1 *v*/*v*) (A) and Methanol: Acetonitrile (25:75) (%) (B)	Testosteron propionate, T. phenyl propionate, T. isocaproate, T. Decanoate Column: Poroshell 120 EC-C18 column (3 mm × 50 mm, 2.7 μm particle size)	100 mg hair obtained now 4 mL of 0.1 M phosphate buffer at pH 9.5 was added to the sample. The sample was then ultrasonicated for 60 min, the extracted with 2 × 4 mL of hexane: ethyl acetate (7:3, *v*/*v*). This was followed by solid phase extraction (SPE) using Bond Elut NH2 cartridges	0.05 pg/mg	0.1 pg/mg	[28]

**Table 2:** Names, structures, and masses of testosterone esters and their respective retention times. Although several esters have similar structures and masses, their retention times differ.

Testosterone and Its Esters	Structure	Molar Mass (g/mol)	[M + H]^+^	Retention Time (min)
Testosterone enanthate (4-Androsten-17β-ol-3-one Enanthate)	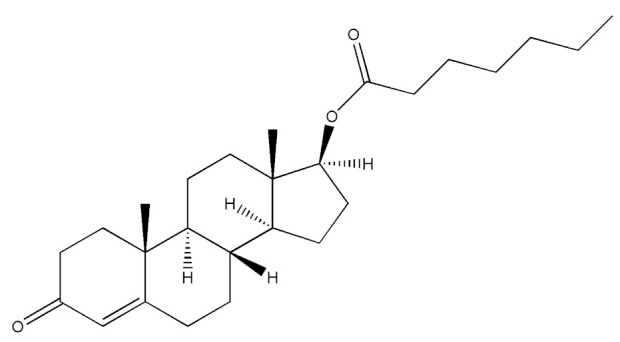	400.59	401.61	0.52
Testosterone benzoate (4-Androsten-17β-ol-3-one Benzoate)	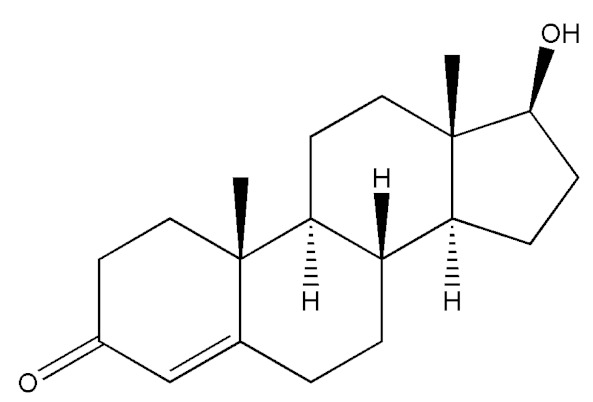	392.53	393.51	3.70
Testosterone cypionate (4-Androsten-17β-ol-3-one Cypionate)	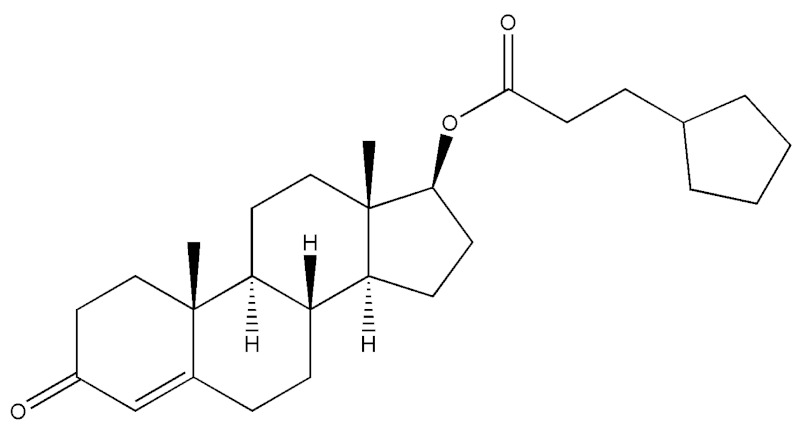	412.60	413.61	3.34
Testosterone decanoate (4-Androsten-17β-ol-3-one Decanoate)	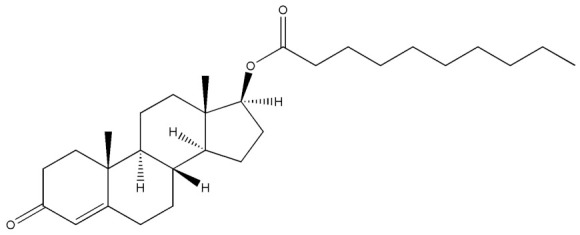	456.70	457.71	5.66
Testosterone valerate	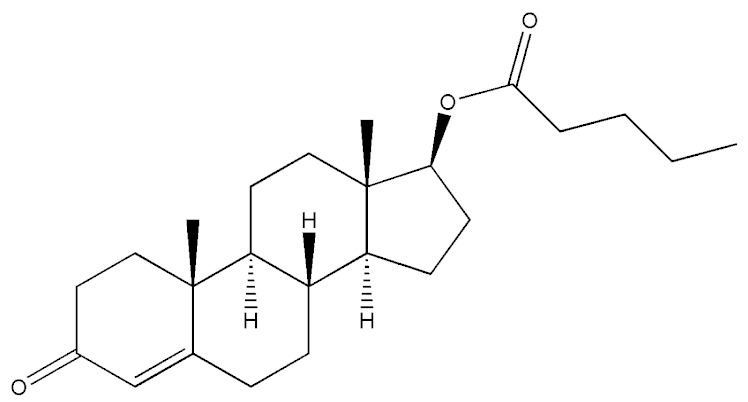	372.50	373.56	3.83
Testosterone caproate	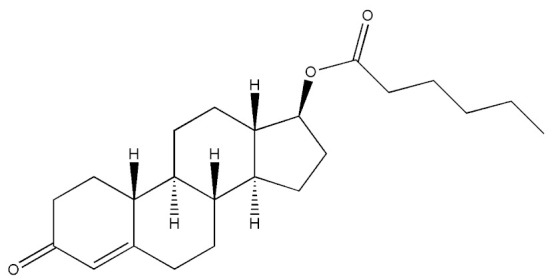	386.60	387.51	3.04
Testosterone (4-Androsten-17β-ol-3-one)	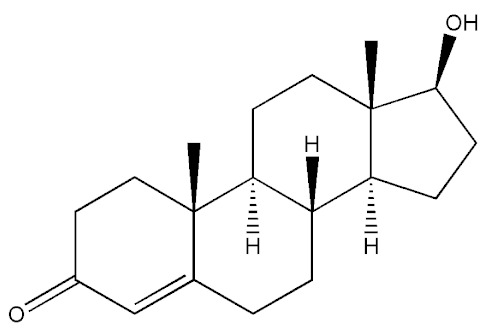	288.41	289.44	1.07
Testosterone laurate	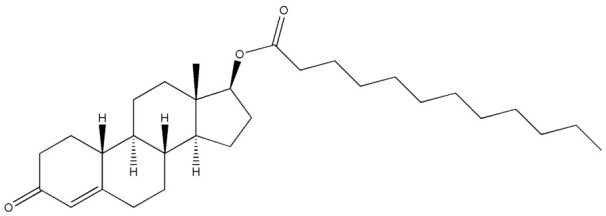	470.73	471.74	6.59
Testosterone undecanoate (4-Androsten-17β-ol-3-one Undecanoate)	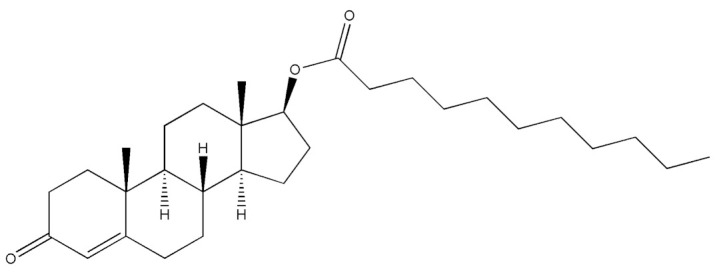	456.70	457.71	6.09
Testosterone isocaproate (4-Androsten-17β-ol-3-one Isocaproate)	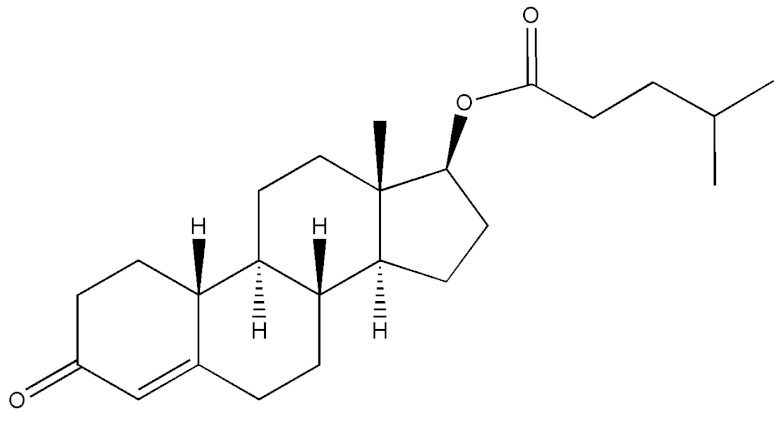	386.58	387.51	4.08
Testosterone hexahydrobenzo-ate (Testosterone 17-cyclohexaanecarboxylate)	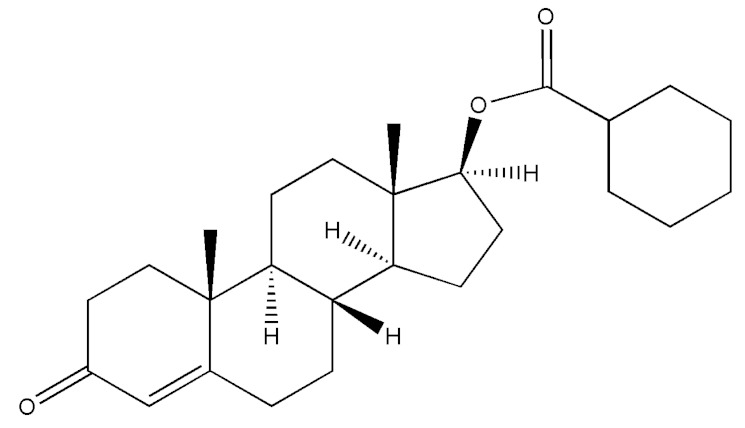	398.58	399.59	4.34

**Table 3:** Analyte names, masses, precursors, product ions, and collision energies.

No.	Analytes	Mass (g/mol)	Precursor (*m*/*z*)	Product (*m*/*z*)	Collision Energy (eV)
1	Testosterone	288.4	289.41	97.1 109 79.2 77.1	21 21 61 77
2	Testosterone enanthate	400.6	401.61	392.8 384.4 89.1	5 9 17
3	Testosterone benzoate	392.5	393.51	77.2 105 97.1 109	85 21 25 41
4	Testosterone isocaproate	386.5	387.51	81.2 96.2 97.1 79.2	33 25 29 73
5	Testosterone cypionate	412.6	413.61	79.1 97.1 238	41 25 21
6	Testosterone laurate	470.73	471.74	97.1 109.1 57.3 81.2	29 29 45 61
7	Testosterone hexahydrobenzoate	398.58	399.59	83.2 231.7	29 93
8	Testosterone valerate	372.55	373.56	97.1 109	29 37
9	Testosterone decanoate	456.70	457.71	97.1 109 95 276.4	37 33 37 13
10	Testosterone capurate	288.43	289.44		
11	Testosterone undecanoate	422.62	443.63	97.1 323.3 252.8	37 25 21

**Table 4:** Comparison between the average concentrations of testosterone esters.

	Camel-Hair Samples	Mean ± SD Conc. (pg/mg)				
N		1	2	3	4	5
		**Valerate**	**Hexahydrobenzoate**	**Decanoate**	**Laurate**	**Testosterone**
1	Sample-01	14.9 ± 0.63				48.5 ± 5.95
2	Sample-02		28.1 ± 6.72			50.8 ± 9.95
3	Sample-03 (Racing camel)		31.8 ± 1.99			48.2 ± 5.11
4	Sample-04 (Racing camel)	11.7 ± 0.50			14.5 ± 2.34	81.2 ± 2.93
5	Sample-05 (Racing camel)			5.1 ± 0.85		169 ± 19.24
6	Sample-06		116 ± 20.2		4.8 ± 0.76	59.2 ± 4.89
7	Sample-07					109 ± 11.84
8	Sample-08					103 ± 13.39
9	Sample-09					18.2 ± 1.28
10	Sample-10					22.8 ± 5.59
11	Sample-11					115 ± 9.36
12	Sample-12					127 ± 21.52
13	Sample-13					91.8 ± 6.53
14	Sample-14					97.6 ± 6.82
15	Sample-15					8.35 ± 1.49
16	Sample-16		151 ± 7.35		15.2 ± 3.84	11.1 ± 2.33
17	Sample-17					44.1 ± 4.55
18	Sample-18				14.13 ± 1.82	15.1 ± 4.02
19	Sample-19	10.5 ± 1.81	12.6 ± 3.24		32.1 ± 1.68	14.7 ± 3.66
20	Sample-20					25.2 ± 9.19
21	Sample-21					119.3 ± 4.31
22	Sample-22					10.7 ± 2.34
23	Sample-23					21.1 ± 3.12
24	Sample-24		55.1 ± 18.5			20.4 ± 1.59

**Table 5:** Method validation parameter values for each QC level.

N	Analytes	Conc. (pg/mg)	Intra-Day			Inter-Day			Recovery (%)
			Precision (% CV)	% Accuracy	SD	Precision (% CV)	% Accuracy	SD	
1	Benzoate	QCH 500	4.4	91.4	20	5.7	96.3	27.6	85.6
		QCM 125	10.3	98.2	12.7	7.9	96.5	9.6	82
		QCL 31.25	7	108.1	2.4	5.8	106.3	1.9	76.7
		QCL-L-3.9	5.5	102.9	0.2	7.9	100.5	0.3	
2	Valerate	QCH 500	2.2	93	10.3	8.2	101.2	41.4	89.4
		QCM 125	11.1	87.6	12.2	8.1	91.6	9.3	96.5
		QCL 31.25	8.5	109	2.9	10.6	102.6	3.4	76
		QCL-L-3.9	3.4	86.5	0.1	12.8	97.7	0.5	
3	Isocaproate	QCH 500	4.8	91.9	22.1	4.2	94	19.9	81.6
		QCM 125	3.8	89.2	4.2	4.1	91.7	4.7	86.9
		QCL 31.25	7.7	109.3	2.6	11.4	117.8	4.2	73.5
		QCL-L-3.9	11.4	104.3	0.5	9.8	103.4	0.4	
4	Hexahydrobenzoate	QCH 500	0.4	88.4	1.8	3.9	96.5	19	88.2
		QCM 125	2.5	89.9	2.8	9.4	97.8	11.5	94.8
		QCL 31.25	4.9	107.8	1.7	18.8	90.8	5.3	79.2
		QCL-L-3.9	3.2	103.9	0.1	2.3	110.2	0.1	
5	Decanoate	QCH 500	2.6	89.6	11.4	1.2	110.8	6.6	88.5
		QCM 125	9.8	98.4	12	4.2	103.8	5.5	82.7
		QCL 31.25	12.1	100.9	3.8	12.8	118.8	4.7	77.2
		QCL-L-3.9	15	94.4	0.6	5	106.2	0.2	
6	Undecanoate	QCH 500	3.8	98.1	18.8	2.9	85	12.3	88.6
		QCM 125	3.8	103.7	4.9	7.1	103.5	9.2	81.9
		QCL 31.25	4.2	104.8	1.4	6.9	102.7	2.2	78.4
		QCL-L-3.9	3.7	97.7	0.1	4.2	106.9	0.2	
7	Laurate	QCH 500	4.3	89	19.3	2.6	105.2	13.4	90.3
		QCM 125	9.1	96	10.9	10.3	89.8	12.7	81.7
		QCL 31.25	7.4	105	2.4	9.1	95.8	2.7	76.7
		QCL-L-3.9	2	18.1	0.1	3.3	106.9	0.1	
8	Testosterone	QCH 500	1.4	107.4	7.6	1.7	88.8	7.5	88.4
		QCM 125	3.7	108.9	5	2.9	105.6	3.9	84.6
		QCL 31.25	6	93.8	1.8	5.5	105.9	1.8	77.2
		QCL-L-3.9	14	97.5	0.5	4.6	108.7	0.2	

## Data Availability

The raw data is available upon request.

## List of Abbreviations

LOD	Limit of Detection
LOQ	Limit of Quantification
LLOQ	Lower Limit of Quantitation
CV	Coefficient of Variation
EIA	Enzyme Immunoassay
°C	Degree Celsius
QCs	Quality Controls
QCH	Quality Control High
QCM	Quality Control Medium
QCL	Quality Control Low
RIA	Radioimmunoassay
SD	Standard Deviation
